# Effects of a 12-Week Change-of-Direction Sprints Training Program on Selected Physical and Physiological Parameters in Professional Basketball Male Players

**DOI:** 10.3390/ijerph17218214

**Published:** 2020-11-06

**Authors:** Seifeddine Brini, Abderraouf Ben Abderrahman, Daniel Boullosa, Anthony C. Hackney, Alessandro Moura Zagatto, Carlo Castagna, Anissa Bouassida, Urs Granacher, Hassane Zouhal

**Affiliations:** 1 Research Unit, Sportive Performance and Physical Rehabilitation, High Institute of Sports and Physical Education of Kef, University of Jendouba, Kef 7100, Tunisia; bseifeddine15@gmail.com (S.B.); bouassida_anissa@yahoo.fr (A.B.); 2 Faculty of Sciences of Bizerte, University of Carthage, Zarzouna, Bizerte 7021, Tunisia; 3 ISSEP Ksar-Essaid, University of La Manouba, Tunis 2000, Tunisia; benabderrahmanabderraouf@yahoo.fr; 4 INISA, Federal University of Mato Grosso do Sul, Campo Grande 79000-000, Brazil; daniel.boullosa@gmail.com; 5 Sport and Exercise Science, James Cook University, Townsville 4811, Australia; 6 Department of Exercise & Sport Science, Department of Nutrition, University of North Carolina, Chapel Hill, NC 27599, USA; thackney@med.unc.edu; 7 Laboratory of Physiology and Human Performance (LAFIDE), Post-Graduation Program in Movement Sciences, Department of Physical Education, São Paulo State University (UNESP), Bauru 17000-000, Brazil; azagatto@yahoo.com.br; 8 University of Rome Tor Vergata, School of Sports and Exercise Science, 00133 Rome, Italy; castagnac@libero.it; 9 Fitness Training and Biomechanics Laboratory, Italian Football Federation (FIGC), Technical Department, 50135 Florence, Italy; 10 Division of Training and Movement Science, University of Potsdam, 14469 Potsdam, Germany; 11 Laboratoire Mouvement, Sport, Santé, University of Rennes, M2S—EA 1274, F-35000 Rennes, France

**Keywords:** jump, fatigue, testosterone, cortisol, team sport, performance

## Abstract

Multidirectional repeated sprints with quick changes-of-direction (CoD) are considered a key performance determinant in basketball. The objective of this study was to investigate the effects of a 12-week CoD sprint training program compared to regular basketball training on selected measures of physical fitness and physiological adaptations in male basketball players. Sixteen professional basketball players were randomly assigned to an intervention group (INT = 8) or an active control group (CON = 8). INT completed a 12-week CoD sprint training program with two sessions per week while CON continued their regular training. Training volume was similar between groups. Before and after the intervention, the two groups were evaluated for the repeated sprint ability test with CoD (IRSA_5COD_), the squat jump (SJ) and countermovement jump (CMJ) test, the five time-jump test (FJT) and change of direction *t*-test. Blood samples were taken before the beginning of the experimental protocol, after 4, 8 and 12 weeks to monitor the testosterone/cortisol ratio (T/C). For *t*-test, post-hoc tests revealed significant pre-to-post improvements for INT (3.4%; *p* = 0.001, ES = 0.91). For CMJ, post-hoc tests revealed a significant pre-to-post decrease for INT (−11.6%; *p* = 0.001, ES = 0.94), and a significant improvement for CON (4.96%; *p* = 0.014, ES = 0.60). For T/C ratio, post-hoc tests revealed a significant decrease after 12 weeks of training for INT (52.3%; *p* < 0.001; ES = 0.63). In conclusion, twelve weeks of CoD sprint training enhanced CoD performance but negatively affected vertical jump capacity in male basketball players. T/C ratio indicated that the physiological demands associated with INT were well-balanced.

## 1. Introduction

Basketball is an intermittent sport which consists of high-intensity efforts interspersed with brief low-intensity periods [1]. High-intensity actions such as jumps, accelerations, decelerations, sprints with quick changes-of-direction (CoD) [2] and the capacity to repeat all these actions are considered key to competitive success [3]. In basketball, 20% of the sprints involve quick CoD [4] which include accelerations and decelerations, together with accelerations in different directions [4]. In order to provide a physical and tactical advantage over the opponent in basketball, it is important to improve movement speed and quick CoD performance [5].

Assuming that sprints with changes-of-direction (CoD) simulates real game-play, previous studies have tried to investigate various protocols and training programs involving CoD sprints in different team sports (i.e., including basketball) [6]. In this context, Attene et al. [7] showed that four weeks of CoD sprint training with two sessions per week enhanced jump performance and aerobic fitness in young male basketball players. Dennis et al. [6] reported that three weeks combined linear sprint and CoD training with two sessions per week significantly improved CoD but not jump performance in highly trained male U15 soccer players. Likewise, Maggioni et al. [8] demonstrated that eight weeks of repeated sprint training with CoD activities did not induce jump performance improvements. Moreover, Morio et al. [9] showed in healthy subjects that jump height decreased significantly in response to CoD activities. Meanwhile, many studies from different intermittent sports have reported that the regular performance of repeated sprints as part of long-term training has the potential to improve aerobic fitness (e.g., VO2max) in the range of 5–10% [10,11]. Taken together, these findings from the available studies on the effects of CoD training or combined CoD and linear sprint training are controversial and the outcomes appear to vary according to the programming parameters applied such as training time, frequency, and intensity. Finally, it is important to note that few studies exist on the effects of CoD sprint training on selected measures of physical fitness and their physiological parameters in professional male basketball players.

Short CoD sprint training incorporates the performance of multiple accelerations and decelerations in different directions, thus potentially generating greater physiological strain [12]. Thus, one can expect that long-term CoD sprint training may negatively affect physical fitness in basketball players and may lead to overtraining. In this context, a wide variety of biochemical, and physiological markers have been used for the monitoring of athletes. In particular, cortisol (C) and testosterone (T) have been identified as reliable markers of training stress responses and can be considered two important hormones in the biochemical assessment of athletes [13,14,15]. In addition, the T/C ratio has additionally been used to evaluate the anabolic/catabolic status in basketball players and other athletes [16,17]. As such, a decline in this ratio has previously been associated with the overtraining syndrome [13,14,15]. Several studies have previously related the reciprocal relation between cortisol and testosterone responses to different exercise programs, including various types of sprint training [16,17,18]. In this context, it has been shown that a brief sprint interval exercise may lead to different anabolic/catabolic and inflammatory responses [19]. These authors [19] have further shown that exercise performed at maximal intensity levels was associated with a significant increase in testosterone, and the T/C ratio. To the best of our knowledge, there are no studies available that assessed the long-term effects of CoD sprint training on T/C ratio in basketball players. Therefore, the aim of this study was to examine the effects of a 12-week CoD sprint training program on selected measures of physical fitness and their physiological correlates in male professional basketball players.

Considering the previous literature [7,13,14,15,16,17,20], we hypothesized that CoD training improves CoD performance, lower limbs muscle power, aerobic fitness and increases the T/C ratio.

## 2. Materials and Methods

### 2.1. Participants

Sixteen professional male basketball players including six guards, six shooting guards, four small forwards (age: 22.06 ± 2.8 years; height, 1.86 ± 0.10 m; body mass, 77.7 ± 7.7 kg; and estimated VO_2max_, 51.0 ± 2.7 mL·min^−1^·kg^−1^) from the second Tunisian division participated in this study. Players exercised between four and five days per week with a daily training time of >60 min, and a training experience of 11.8 ± 3.9 years. Athletes were randomly assigned according to their playing position to the intervention (INT, *n* = 8) or the active control group (CON, *n* = 8). Each group included three guards, three shooting guards, and two small forwards.

This study was conducted during the competitive season and it was approved by the Clinical Research Ethics Committee of the High Institute of Sports and Physical Education of Kef, University of Jendouba, Kef, Tunisia (approval No. 8/2017). The experimental protocol was conducted according to the latest version of the Declaration of Helsinki. All participants provided their written informed consent before study participation.

### 2.2. Procedures

Players were familiarized with all tests and procedures before the start of the experimental protocol. To allow sufficient recovery before testing, the last training session was scheduled 48 h prior to testing. To minimize any effects of diurnal variations, the two testing sessions were conducted ±2 h of the same time of day. Players were instructed to wear the same footwear during all test sessions. Overall, the study lasted 15 weeks and was conducted during the 2017/2018 season. The experimental period started in October 2017 and lasted until January 2018.

During the competitive season, participants exercised five times per week between 6:00–7:30 p.m. and completed one game per week on the weekend. INT completed a 12-week CoD sprints training program (see details below) with a frequency of two sessions per week (Tuesday and Thursday), while the active CON completed an intensive transition drill or a simulated game (intensity (rate of perceived exertion, RPE): ~7; volume = 3 sets of 4 min with 2 min recovery) over the course of the study period. The session rating of perceived exertion was evaluated immediately at the end of each session using the Borg CR-10 scale [21] in order to assess internal training load of all the sessions. Players were fully familiarized with this scale, which was regularly used during their sports season.

In both groups, training sessions started with a 15-min warm-up program consisting of 5 min of low-intensity running, 5 min of dynamic stretching, and 5 min of skipping exercises followed by technical and tactical drills based on basic basketball movements (i.e., offensive, ready stance, running, CoD, linear sprint, stopping, pivoting, and jumping exercises), specific basketball movements (triple threat position, pivot, face up or one- and two-phase stop), basketball technique fundamentals (dribbling, passing, and shooting), basic defensive movements (defensive stance, defensive slide, denial defense, and box-out) and a simulated game at the end of every session, which were identical for both groups. Both groups completed the same training volume (~90 min per session) over the course of the study.

Before (T1) and after (T2) the intervention, the two groups performed the following tests in random order: a repeated sprint ability test with five CoD (IRSA_5COD_ test) [22], the squat jump (SJ) [23] and countermovement jump (CMJ) test [23], the five time jump test (FJT) [24], the *t*-test [25], and the Yo-Yo intermittent recovery level 1 (Yo-Yo IR1) test [26]. The testing sessions were scheduled over a period of one week with 48 h between test sessions.

In order to control the variation of hormones, blood samples were always taken before the beginning of the experimental period (P1), after four weeks (P2), eight weeks (P3), and twelve weeks (P4) to determine the testosterone/cortisol ratio.

### 2.3. Change-of-Direction Sprint Training

**Figure 1:**
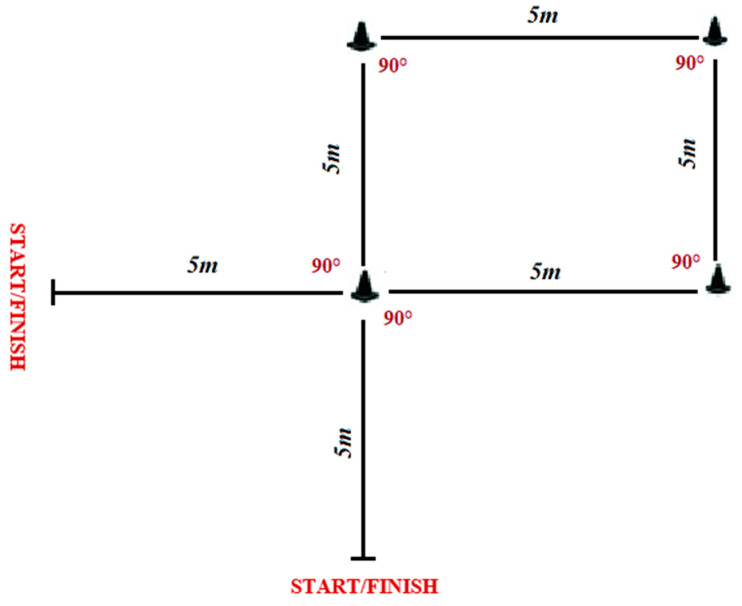
Schematic description of repeated sprint training with change of direction.

INT completed 12 weeks of CoD sprint training with a frequency of two sessions per week. Each training session consisted of the following sequences: (i) briefing with coaches and organization of the training session (10 min); (ii) warm-up (15 min consisting of 5 min of low-intensity running, 5 min of dynamic stretching, and 5 min of skipping exercises); (iii) the exercise intervention (CoD training) (20 min); (iv) technical/tactical exercises to get prepared for the weekend match (30 min); (v) cool-down consisting of light running (10 min). Overall, a single training session lasted 90 min. Moreover, players had 15 min before and after the training session to change and take a shower. Training interventions consisted of three sets with eight repetitions each over 30 m (6 × 5 m) sprint distances at maximal intensity (including 90° CoD) (Figure 1) with 20 s of passive recovery between the repetitions and a 4 min rest between sets. Participants were encouraged to sprint at maximal effort during each sprint.

### 2.4. Assessment

#### 2.4.1. Anthropometrics and Maximal Oxygen Consumption

Body mass (kg) was measured with an electronic scale (Pharo 200 Analytic, Hamburg, Germany) and height (m) with a portable stadiometer (Seca, Maresten, UK). Maximal oxygen consumption (VO_2max_) was estimated using the 20 m shuttle run test according to the Léger and Gadoury equation [27].

#### 2.4.2. *t*-Test

The CoD *t*-test is a valid test to evaluate CoD performances in basketball as it includes forward, lateral, and backward running over short distances [25]. Two trials were completed and the fastest trial was taken for further analysis. Times were recorded to the nearest 0.01 s using an electronic timing system (Brower Timing Systems, Salt Lake City, UT, USA) placed 0.4 m above the ground.

#### 2.4.3. Yo-Yo Test Level 1

The Yo-Yo test consisted of 20 m shuttle runs performed at increasing velocities with 10 s of active recovery between the shuttle runs until exhaustion [26]. Audio cues of the Yo-Yo IR1 test were played on a CD player (Philips, Az1030 CD player, Eindhoven, Holland). The end of the test was considered when the participant failed twice to reach the front line in time or was unable to complete another shuttle at the dictated speed. The total distance (TD) covered during the Yo-Yo IR1 was recorded for comparisons.

#### 2.4.4. Intensive Repeated Sprint Ability Test (IRSA_5COD_)

Intensive repeated sprint ability test (IRSA_5COD_) was used to assess players’ ability to cope with the intermittent demands of basketball [22]. This test includes rapid CoD, which represents sport-specific movement patterns in basketball. Players completed 10 × 30 m maximal sprints (6 × 5 m) interspersed with 30 s of passive recovery. Sprint times were recorded with photocells (Brower timing system, Salt Lake City, UT, USA; accuracy of 0.01 s). The participants started from the starting line and accelerated as fast as possible and sprinted over a distance of 5 m. There, they decelerated and touched the second line with one foot, turned around and ran back to the starting line, before they turned right, accelerated and sprinted over 5 m, and touched the third line with their foot. Next, they turned around and ran back to the starting line where they turned right, accelerated and sprinted for 5 m and touched the fourth line with their foot, turned around, accelerated and sprinted back to the start/finish line (i.e., along a “T” letter shaped circuit) [22].

During the test, heart rate frequency was continuously recorded using a cardio-frequency monitor (Polar Electory, Kempte, Finland).

The rating of perceived exertion (RPE) was assessed immediately following each IRSA_5COD_ test using a Borg’s CR-10 scale [21]. Players were familiarized with this scale, which was regularly used during the season.

The maximal blood lactate concentration (mmol·L^−1^) was measured from capillary blood samples obtained from the earlobe at the third minute after the end of the IRSA_5COD_ test [28]. The blood sample was immediately analyzed using a portable lactate analyzer (Arkray Lactate Pro LT-1710 Kyoto, Japan) previously calibrated following the manufacturer’s instructions.

#### 2.4.5. Vertical Jump Tests

Vertical jumps height was evaluated using an optoelectric system (Opto-Jump Microgate—Italy). Jump height was calculated according to the following equation: jump height = 1/8 × g × t^2^, where g is the acceleration due to gravity and t is the flight time [29]. Players performed the countermovement (CMJ) and the squat jumps (SJ) according to the previously described protocols [23]. Before testing, players performed submaximal CMJs and SJ (2 repetitions each) after the warm-up. Each participant performed 3 maximal CMJs and SJs, with approximately 2 min of recovery in between. Players were asked to jump as high as possible. The best trial was used for further analyses.

#### 2.4.6. Five-Time Jump Test (FJT)

The FJT test is a practical and valid test and is often used as a proxy for lower limbs muscle power [24]. At the beginning of the test and after the fifth jump, feet are in parallel position. FJT performance was recorded in meters (m) to the nearest cm. Participants performed two trials and the best trial was used for further analyses.

#### 2.4.7. Blood Samples

Blood samples were collected in fasted state from the antecubital vein every fourth week at the same time of day (between 09.30–10.00 a.m.) for plasma cortisol and testosterone concentration measurements. Blood collection was performed 48 h after the last intensive training session on Thursdays. Blood serum was stored at −20 °C until analyses. The plasma cortisol and testosterone concentrations were measured by an automated analyzer (Vidas Biomerieux, France) using the ELFA technique (Enzyme linked-Fluorescent-Assay) with validated kits (VIDAS^®^ cortisol 13414 F; VIDAS^®^ testosterone 9307142 D).

### 2.5. Statistical Analysis

Data are presented as means and standard deviations (SD). Normality of data was tested and confirmed using the Shapiro–Wilk test, baseline between group differences were computed using *t*-tests for independent samples.

An a priori power analysis (*N.B.,* expected SD of residuals = 2 cm for CMJ and SJ, desired power = 0.80, and alpha error = 0.05) was computed to simulate a statistically significant group-by-time interaction effect for our primary outcome of jump performance [30]. The analysis revealed that a sample size of *n* = 8 per group would be sufficient to achieve medium-sized group-by-time interactions.

The effects of training were evaluated using a 2 (groups: INT, CON) × 2 (time: Pre-test, Post-test) mixed model ANOVA. If a statistically significant interaction effect was found, Bonferroni corrected post-hoc tests were calculated.

Additionally, effect sizes (ES) were determined from ANOVA output by converting partial eta-squared to Cohen’s d. In addition, within-group ES were computed using the following equation: ES = (mean post—mean pre)/SD [31]. Following [32], ES were considered trivial (<0.2), small (0.2–0.6), moderate (0.6–1.2), large (1.2–2.0) and very large (2.0–4.0). Additionally, intraclass correlation coefficients (ICC) and coefficients of variation (CV) were computed to assess relative and absolute test–retest reliability (see Table 1). ICCs were classified as ICC < 0.50 weak, 0.50 to 0.79 moderate, and ≥0.80 strong. The level of significance was set at *p* < 0.05. All statistical analyses were computed using SPSS for Windows, version 18.0 (SPSS Inc., Chicago, IL, USA).

## 3. Results

All players from both groups completed the study according to the previously described methodology. No injuries occurred over the course of the study. During the 12-week intervention period, adherence rates were 94.1% for INT and 92.3% for CON. The average playing time per game was 28.3 ± 1.6 min for INT and 27.7 ± 1.6 min for CON. No statistically significant between-group differences were observed for these measures. In addition, no significant between-group baseline differences were found for any of the analyzed parameters (see Table 2 and Table 3). Table 2 illustrates performance and physiological parameters during the IRSA_5COD_ test. The statistical analyses revealed no significant main effects for time, group, and group x time interactions for total time (TT), best time (BT), fatigue index (FI), heart rate (HR), lactate concentration [Lac] and rating of perceived exertion (RPE).

Table 3 shows the findings for the jump tests, the *t*-test, and the Yo-Yo IR1. Significant main effects for time were observed for SJ, CMJ, and FJT as well as for the *t*-test and the Yo-Yo IR1 (*p* = 0.001, ES = 0.60, moderate; *p* = 0.003, ES = 0.50, small, respectively). A significant group × time interaction was found for *t*-test performances (*p* = 0.003, ES = 0.74, moderate). Bonferroni corrected post-hoc test revealed significant pre-to-post improvements for INT (3.38%; *p* = 0.001, ES = 0.91, moderate). Moreover, a significant group × time interaction was observed for CMJ height (*p* = 0.001, ES = 0.92, moderate). Bonferroni corrected post-hoc tests revealed a significant pre-to-post decrease for INT (11.58%; *p* = 0.001, ES = 0.94, moderate), and a significant improvement for CON (−4.96%; *p* = 0.014, ES = 0.60, moderate).

**Figure 2:**
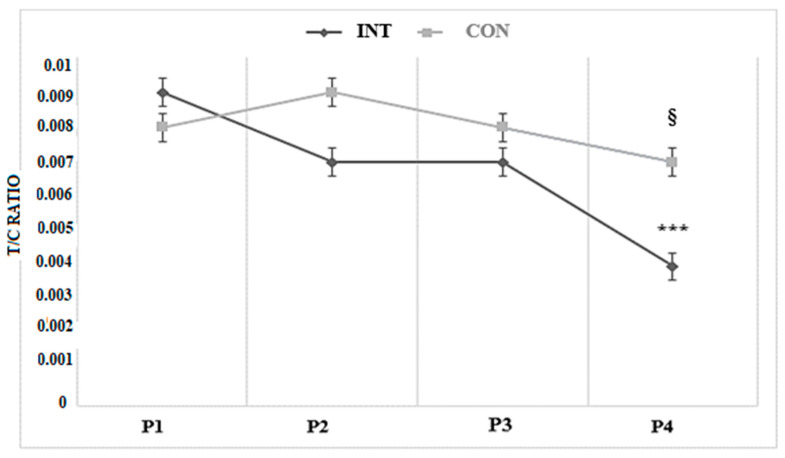
Variation of testosterone/cortisol ratio during the experimental period for the intervention and active control groups. Legend. INT: intervention group; CONT: active control group; T/C RATIO: testosterone/cortisol ratio; P1: blood samples before the beginning of the experimental period; P2: after 4 weeks; P3: after 8 weeks; P4: after 12 weeks; ***: significant differences between blood sampling at P4 *p* < 0.001; §: significant differences between groups at P4 *p* < 0.01.

Variations of the testosterone/cortisol ratio are presented in Figure 2. The analysis revealed a significant main effect for time for T/C ratio (*p* = 0.001, ES = 0.95, moderate). In addition, a significant group × time interaction was identified for T/C ratio (*p* = 0.02, ES = 0.82, moderate). Bonferroni corrected post-hoc test showed a significant decrease after 12 weeks of training for INT (52.29%; *p* < 0.001; ES = 0.63, moderate).

## 4. Discussion

The objective of this study was to examine the long-term effects of CoD sprint training on selected measures of physical fitness and their physiological correlates in healthy male professional basketball players. The main results of the present study manifested on significant interactions between group-by-time effect on CoD, CMJ and testosterone/cortisol ratio. In fact, 12 weeks of CoD sprint training enhanced CoD and aerobic fitness in male professional basketball players which is in support of our study hypothesis. However, jump performances and T/C ratio showed significant decreases following CoD sprint training which is why we had to reject our study hypothesis.

Performance indices in the IRSA_5COD_ did not change significantly after 12 weeks of training in both groups (INT and CONT). Regarding TT and BT, it could be argued that this result may be attributed to the shorter distance of sprints (5 m) during the IRSA_5COD_ which was different from the previous repeated sprint protocols used by our team (15 + 15 m and 10 + 10 + 10 m), with important differences on mechanical and metabolic demands. In fact, because of this short distance used during the IRSA_5COD_, players were not able to reach their maximum speed. Regarding FI, the absence of change may be explained by its low reproducibility [33]. Meanwhile, the usefulness of this index for a coach is still under debate as a better FI does not necessarily indicate better RSA performances [34].

Concerning jump performances, our results showed that 12 weeks of CoD sprints training led to a significant decrease for the INT group. However, the CONT group exhibited a significant improvement in CMJ after this training period. In this study, we observed a decline in jump performance after CoD but not linear sprint training. Long-term CoD training compared with linear sprint training might be physically more demanding which therefore may induce greater physical stress in the form of fatigue. This is in line with results from Mario et al. [9] who examined the effects of intermittent exhaustive rebound exercise on the time course of neuro-mechanical changes underlying the stretch–shortening cycle in healthy subjects. These authors observed that jump height decreased with the regular performance of CoD activities. However, more research is needed that substantiates our preliminary findings on compromised jump performance following CoD training. Moreover, our findings could be justified by the decrease in the T/C ratio at the end of the training program in the INT group, which is suggestive of overtraining [13,35]. Consequently, this catabolic state at the end of the training program could be also related to the decrease in jump performances and associated lower limbs explosiveness, thus also limiting the expected improvement in the IRSA_5COD_. Further studies should verify if a lower dose (e.g., one day of CoD sprint training a week) could be sufficient to improve specific performances without these unexpected negative adaptations. Our findings disagree with previous studies reporting significant improvement in jump performances of young basketball players completing a shorter training period (i.e., four weeks) but with a different training program [7]. Otherwise, another previous study reported no significant changes in SJ and CMJ after eight weeks of an RSA training program with one CoD [8]. This significant decrease in both vertical and horizontal performances in INT is somewhat unsurprising since it confirms the evidence suggesting that sprinting with multiple changes of direction involves specific motor qualities different from other RSA protocols without CoD, but with a greater physiological strain [12]. In fact, due to the specific design of the exercise with multiple CoD, it was expected to increase the number of accelerations and decelerations in different directions when compared to a traditional RSA exercise.

Our results revealed that CoD sprint training was not more effective than the regular training to improve the Yo-YoIR1 performance. In fact, both groups (INT and CON) showed a significant comparable improvement in total distance covered in the Yo-YoIR1. Concerning the improvement of the aerobic fitness for INT group, our results are in agreement with previous investigations that reported improvements in aerobic fitness after RSA training [8]. Previously one study [10] described a significant improvement in peak oxygen uptake (10–12%) after five weeks of repeated sprint training (i.e., 5 × 6 s sprints, every 30 s, three days/week).

Similar results were also described by another study [11] in which a significant improvement in maximal oxygen uptake (i.e., 6.6%) was observed after 12 weeks of training using three sets of 6 × 40 m sprints interspaced by 20 s of rest. In addition, another study [7] reported a significant increase in the aerobic fitness after four weeks of repeated CoD training in young male basketball players. Meanwhile, given the mixed demands (aerobic and anaerobic) of the Yo-Yo IR1 test, it remains to be elucidated in further studies if a higher glycolytic capacity may be also related to these improvements. Otherwise, the decrease in T/C ratio after CoD training negatively affected jump performance without having any negative impact on aerobic performance. In fact, the decline in the T/C ratio at the end of the experimental period was due to a decrease in testosterone levels only. In this context, it has been shown that testosterone concentrations correlate with CMJ performance [36]. For example, Moreira et al. [37] reported that following division of the groups according to low or high testosterone concentrations, a significant difference between groups was found regarding CMJ but not Yo-YoIR1 performance in elite young Brazilian soccer players. Moreover, those authors explained that the contribution of testosterone variance in Yo-YoIR1 was lower for the whole group than that verified through CMJ data. This latter point could argue that the higher contribution from testosterone levels to CMJ performance might be related to it having a more important role in strength/power-related performance compared with intermittent endurance type activities. Hence, the findings of this study could explain and support our results.

Finally, our results showed a significantly better CoD performance in favor of INT group in comparison with CONT group which was in accordance with previous investigations that recorded a significant improvement of CoD performance following repeated sprints training with CoD [6,9]. Our results could be explained by the similarity between the IRSA_5COD_ and the *t*-test in design and demands thus allowing to a better CoD performance. However, the fact that only the *t*-test exhibited significant differences, could be indicative of this test being a pure CoD test, while the IRSA_5COD_ also requires lower limbs explosiveness and power which were worsened after the training program.

### Limitations

Our study has some limitations. The first being the small sample size, which reduces our statistical power. Secondly, we examined only male basketball players and it is necessary that future works examine the responses of female basketball players too. Finally, our study did not include a test of retention; therefore, we do not know for how long the performance gains lasted after this type of training program.

## 5. Conclusions

Our results showed that 12 weeks of CoD sprint training with two sessions per week significantly enhanced CoD in professional basketball players. Moreover, our results revealed that CoD training was not more effective than regular training to improve aerobic performance. Following CoD training, jump performance declined. However, the T/C ratio indicated that the physiological demands associated with INT were well-balanced.

### Practical Applications

This study indicated that a long-term CoD sprint training program improved aerobic capacity and CoD performance in male senior basketball players. Therefore, we recommend coaches to adopt this training program in order to improve these qualities over the competitive season. However, coaches should be careful with regards to the total load of the training sessions to avoid excessive fatigue which could negatively affect jump performances and the muscle power of lower limbs. In this context, the regular monitoring of the T/C ratio could be useful to avoid this maladaptation.

## Figures and Tables

**Table 1:** Intraclass correlation coefficients (ICCs) for relative reliability and coefficients of variation for absolute reliability of the applied physical fitness tests.

Measures	ICC	95% CI	% CV
IRSA_5COD_	0.92	0.77–0.98	4.1
Squat jump	0.96	0.79–0.98	4.6
Countermovement jump	0.97	0.86–0.97	4.4
Five-time jump test	0.98	0.88–0.98	3.1
*t*-test	0.98	0.92–0.99	4.3
Yo-Yo IR1	0.93	0.73–0.95	2.3
Testosterone	0.94	0.83–0.94	3.6
Cortisol	0.96	0.77–0.96	4.1

Note: ICC—intraclass correlation coefficient; CI—confidence interval; CV—coefficient of variation (%). IRSA_5COD_, repeated sprint ability test with five changes of direction (CoD); Yo-Yo IR1, Yo-Yo intermittent recovery level.

**Table 2:** Intensive repeated sprint ability test performances and physiological parameters determined before (pre-test) and after (post-test) the change-of-direction sprint training program for the intervention and the active control group.

							*p*-Values (Effect Size)		
IRSA_5COD_ Test	INT (*n* = 8)			CON (*n* = 8)			Time	Group	Group × Time
	Pre Test	Post Test	Δ%	Pre Test	Post Test	Δ%			
TT (s)	70.09 ± 0.98	70.05 ± 0.99	0.04 ± 0.05	69.81 ± 0.62	69.75 ± 0.63	0.08 ± 0.07	0.27 (0.15)	0.11 (0.31)	0.41 (0.09)
BT (s)	6.91 ± 0.09	6.90 ± 0.10	0.16 ± 0.35	6.87 ± 0.12	6.90 ± 0.11	−0.42 ± 0.84	0.50 (0.07)	0.06 (0.42)	0.07 (0.39)
FI (%)	1.26 ± 0.58	1.15 ± 0.50	8.57 ± 17.76	1.19 ± 0.63	1.03 ± 0.59	14.56 ± 23.59	0.12 (0.29)	0.26 (0.17)	0.65 (0.03)
HR (beat/min)	186.75 ± 2.77	187.15 ± 3.47	−0.22 ± 1.61	186.90 ± 5.75	188.05 ± 3.75	−0.65 ± 1.38	0.30 (0.15)	0.78 (0.01)	0.61 (0.04)
Lac (mmol/l)	5.37 ± 2.06	5.25 ± 2.66	−14.43 ± 80.63	5.75 ± 2.43	6 ± 2.93	−12.36 ± 48.83	0.94 (0.001)	0.50 (0.05)	0.77 (0.13)
RPE	5.62 ± 1.40	5.75 ± 0.88	−3.33 ± 18.77	6 ± 1.30	5.76 ±1.03	3.86 ± 11.44	0.97 (0.003)	0.56 (0.07)	0.81 (0.16)

Note: Data are means and standard deviations. TT: total time; BT: best time; FI: fatigue index; HR: heart rate; Lac: lactate concentration; RPE: rating of perceived exertion.

**Table 3:** Jump, change-of-directions and Yo-Yo intermittent recovery test level 1 performances determined before (pre-test) and after (post-test) the change-of-direction sprint training program for the intervention and the active control group.

							*p*-Value (Effect Size)		
Variables	INT (*n* = 8)			CON (*n* = 8)			Time	Group	Group × Time
	Pre Test	Post Test	Δ%	Pre Test	Post Test	Δ%			
SJ (cm)	40.25 ± 6.58	35.13 ± 4.01 *	11.68 ± 10.21	42 ± 6.41	36.37 ± 5.07 **	13.16 ± 4.80	0.002 (0.77)	0.31 (0.15)	0.75 (0.02)
CMJ (cm)	43.87 ± 6.88	39 ± 7.76 ^†^ ***	11.58 ± 4.30	40.25 ± 6.98	42.12 ±6.91 **	−4.96 ± 4.79	0.006 (0.68)	0.84 (0.007)	0.001 (0.92)
FJT (m)	7.37 ± 0.46	7.26 ± 0.44 ***	1.54 ± 0.85	7.40 ± 0.56	7.35 ±0.59	0.78 ± 1.30	0.006 (0.70)	0.37 (0.12)	0.15 (0.27)
*t*-test (s)	10.21 ± 0.90	9.86 ± 0.91 ^††^ ***	3.38 ± 1.13	10.18 ±0.98	10.01 ± 0.87 **	1.70 ± 1.24	0.001 (0.60)	0.016 (0.6)	0.003 (0.74)
YO-YOIR1 (m)	1792.50 ± 208.65	2065 ± 331.18 **	−14.89 ± 11.03	1627.50 ± 412.78	1805 ± 529.66 **	−10 ± 6.76	0.003 (0.50)	0.13 (0.29)	0.24 (0.19)

Note: Data are means and standard deviations. SJ: squad jump test, CMJ: countermovement jump test, FJT: five jump test; *t*-test: CoD T test, Yo-Yo IR1: Yo-Yo intermittent recovery test level 1. * Significantly different between Pre- and Post-test, *p* < 0.05; ** significantly different between Pre- and Post-test, *p* < 0.01; *** significantly different between Pre- and Post-test, *p* < 0.001; ^†^ significantly different between INT and CON, *p* < 0.05; ^†^^†^ significantly different between INT and CON, *p* < 0.01.
